# Reduced Levels of Misfolded and Aggregated Mutant p53 by Proteostatic Activation

**DOI:** 10.3390/cells12060960

**Published:** 2023-03-21

**Authors:** Evelyne Naus, Marleen Derweduwe, Youlia Lampi, Annelies Claeys, Jarne Pauwels, Tobias Langenberg, Filip Claes, Jie Xu, Veerle Haemels, Zeynep Kalender Atak, Rob van der Kant, Joost Van Durme, Greet De Baets, Keith L. Ligon, Mark Fiers, Kris Gevaert, Stein Aerts, Frederic Rousseau, Joost Schymkowitz, Frederik De Smet

**Affiliations:** 1 VIB-KU Leuven Center for Brain & Disease Research, Herestraat 49, 3000 Leuven, Belgium; evelynenaus@gmail.com (E.N.); youlia.lampi@kuleuven.vib.be (Y.L.); tobias.langenberg@vib.be (T.L.); filip.claes@aelintx.com (F.C.); xujieletter@gmail.com (J.X.); zeynep.kalender@gmail.com (Z.K.A.); rob.vanderkant@kuleuven.be (R.v.d.K.); jvdurme@gmail.com (J.V.D.); grdbaets@gmail.com (G.D.B.); mark.fiers@kuleuven.be (M.F.); stein.aerts@kuleuven.be (S.A.); frederic.rousseau@kuleuven.be (F.R.); joost.schymkowitz@kuleuven.be (J.S.); 2 Switch Laboratory, Department for Cellular and Molecular Medicine, Katholieke Universiteit Leuven, 3000 Leuven, Belgium; 3 The Laboratory for Precision Cancer Medicine, Translational Cell and Tissue Research Unit, Department of Imaging and Pathology, Katholieke Universiteit Leuven, 3000 Leuven, Belgiumveerle.haemels@gmail.com (V.H.); kris.gevaert@vib-ugent.be (K.G.); 4 VIB-UGent Center for Medical Biotechnology, 9052 Ghent, Belgium; jarne.pauwels@vib-ugent.be; 5 Department of Biomolecular Medicine, Ghent University, 9052 Ghent, Belgium; 6 Laboratory of Computational Biology, Center for Human Genetics, Katholieke Universiteit Leuven, 3000 Leuven, Belgium; 7 Department of Medical Oncology, Center for Molecular Oncologic Pathology, Dana-Farber Cancer Institute, Boston, MA 02215, USA; keith_ligon@dfci.harvard.edu; 8 The Broad Institute, Cambridge, MA 02142, USA; 9 Department of Pathology, Division of Neuropathology, Brigham and Women’s Hospital and Children’s Hospital Boston, Boston, MA 02215, USA; 10 Department of Pathology, Harvard Medical School, Boston, MA 02215, USA

**Keywords:** protein aggregation, p53, heat-shock, proteostasic modulation

## Abstract

In malignant cancer, excessive amounts of mutant p53 often lead to its aggregation, a feature that was recently identified as druggable. Here, we describe that induction of a heat shock-related stress response mediated by Foldlin, a small-molecule tool compound, reduces the protein levels of misfolded/aggregated mutant p53, while contact mutants or wild-type p53 remain largely unaffected. Foldlin also prevented the formation of stress-induced p53 nuclear inclusion bodies. Despite our inability to identify a specific molecular target, Foldlin also reduced protein levels of aggregating SOD1 variants. Finally, by screening a library of 778 FDA-approved compounds for their ability to reduce misfolded mutant p53, we identified the proteasome inhibitor Bortezomib with similar cellular effects as Foldlin. Overall, the induction of a cellular heat shock response seems to be an effective strategy to deal with pathological protein aggregation. It remains to be seen however, how this strategy can be translated to a clinical setting.

## 1. Introduction

The stabilization and excessive accumulation of mutant p53 (mutp53) in tumor cells is a hallmark of malignancy [1]. This event forms a prerequisite for p53 gain-of-function activity, which has been confirmed in several mouse models, as well as in Li-Fraumeni patients carrying germline p53 mutations, where the presence of mutp53—as opposed to a complete genetic deletion and absence of the protein—causes a worsened tumorigenic profile [2,3]. Mutations in p53 are generally subdivided into either contact mutations (mutp53^CON^) that impair the DNA-binding interface but retain p53′s native fold, or structurally destabilizing mutations (mutp53^STRUC^) that affect the thermodynamic stability of the p53 DNA-binding domain, resulting in unfolding and misfolding of the protein [4] and, as a consequence, impair DNA binding and its transcriptional activity. Previous work shows that the accumulation of mutp53 can also lead to its aggregation and coaggregation with other proteins in tumor cells [5,6,7,8], which is associated with lower disease-free and overall patient survival in cohorts of glioblastoma and colon and ovarian cancer [9,10]. Thus, contrary to the cytotoxic effects of protein aggregates in archetypical aggregation pathologies such as Alzheimer’s, Huntington’s, amyotrophic lateral sclerosis (ALS) and Parkinson’s disease, protein aggregates in cancer cells can contribute to tumor maintenance and progression [11]. 

Due to its important tumor suppressor function, most p53-based therapeutic approaches have focused on either exploiting or enhancing residual p53 functionality or on restoring tumor-suppressor activity with mutp53, rather than removing accumulated/aggregated mutp53 [12]. However, it was also shown that mutp53 has potential as an actionable drug target by showing that the tamoxifen-induced ablation of mutp53 in allotransplanted and endogenous murine tumors resulted in tumor cell apoptosis and extended animal survival [13]. In addition, it has been shown that the excessive accumulation of mutp53 is enhanced by an interaction with HSP90 [14,15] and that the inhibition of HSP90 resulted in p53 degradation and tumor apoptosis [12,13]. More recently, molecules that directly interfere with p53 aggregation were identified; these include a small peptide (ReACp53) and the novel synthetic amyloid blocker ADH6 [16], which both interfere with the aggregation-prone region that our group previously identified in the DNA-binding domain of p53 [7,17]. In addition, two rather non-specific molecules, resveratrol and emodin [18,19,20], have also been identified as molecules that could indirectly interfere with p53 aggregation. While the clinical translatability of these inhibitors remains to be uncovered, resveratrol has been described as a molecule with broad spectrum effects on cellular homeostasis potentially having an indirect effect on the reactivation of p53, in addition to having the potential to deal with mildly aggregating forms of p53 [20]. The effect of emodin on p53 aggregation was mainly attributed to the autophagy-mediated removal of aggregates, even though with low efficiency [19]. Finally, the thermodynamic stabilization of the specific p53 Y220C mutant by PhiKan molecules [21] was shown to slow down p53′s aggregation trajectory, providing additional insights into the aggregation mechanisms and kinetics, but it remains to be seen how generalizable this approach is towards other, more prevalent p53 mutants. Overall, these findings provide support for the therapeutic value of dealing with accumulated/aggregated mutp53.

Parallel to cancer research, a major research focus into amyloid diseases consists of identifying misfolded and/or aggregated protein species displaying a toxic gain-of-function [22] and how these are dealt with by the proteostatic machinery [23]. First, next to cancer, also in AD brains, aggregated p53 species have been identified in conjunction with Tau and Aß [24]. Moreover, in neurodegenerative diseases, HSP90 inhibition enables the cell to process misfolded proteins but not pre-formed aggregates, while the degradation of the latter can be improved by enhancing HSP70 activity [25,26]. In Parkinson’s, the overexpression of HSP70 reduces high molecular weight, aggregated alpha-3-synuclein species and toxicity in vivo [27]. If the same holds true for mutp53 in cancer, HSP70 activation could also enhance the processing of misfolded/aggregated mutp53. Sufficient precaution has to be taken, however, as HSP70 chaperone members have also been linked to tumor maintenance and mutp53 can be stabilized by canonical HSP70 family members, such as HSPA1A [15]. On the other hand, the theory of exploiting HSP70 for mutp53 degradation is supported by the observation that HSF-1 induces differential transcriptional profiles in malignancy compared to those with physical heat stress [28]. 

In this study, we aimed to specifically remove accumulated levels of aggregated, mutp53 from cancer cell lines by activating the heat shock response machinery. As such, we found that the induction of a heat shock-related stress response by exposure to the tool compound Foldlin resulted in significantly reduced protein levels of mutp53^STRUC^ in conventional and primary cancer cell lines, while leaving natively folded p53 unaffected. Despite our inability to identify a specific molecular target for Foldlin, exposure to Foldlin also reduced protein levels of aggregating SOD1 variants. In addition, we performed a phenotypic screen using a library of 778 FDA-approved small molecules for their ability to reduce misfolded mutant p53 protein levels while increasing HSP70 levels in cancer cells. Here, we found that the proteasome inhibitor Bortezomib exhibited similar cellular effects as Foldlin. Overall, our data suggest that the induction of a cellular heat shock response is an effective strategy to deal with pathological protein aggregation in vitro, although it remains to be uncovered whether this can also be achieved in an in vivo setting.

## 2. Materials and Methods

### 2.1. Compounds

Foldlin was identified in a screen comprising approximately 900,000 small molecules that induce an HSP70 response in HeLa cells^38^. The compound was resynthesized by EcoSynth (Oostende, Belgium). See Supplementary Information on the synthesis and quality control in Supplementary Figure S1. The inactive molecule was kindly provided by Proteostasis Therapeutics Inc (Boston, MA, USA). The SCREEN-WELL^®^ FDA approved drug library V2 was obtained from Enzo life sciences (Farmingdale, NY, USA). ML346 (HY-18669) and KRIBB11 (HY-100872) were obtained from MedChemExpress (Monmouth Junction, NJ, USA).

### 2.2. Cell Culture

Cancer cell lines (as described in Table 1) were cultured in a humidified atmosphere containing 5% CO_2_ at 37 °C. Cells were obtained from the American Type Culture Collection (ATCC) and were grown according to the supplier’s instruction in medium (DMEM) supplemented with 10% FCS (GIBCO), non-essential amino acids and glutamine (Life Technologies, Carlsbad, CA, USA). The primary GBM cultures were grown in NeuroCult medium as previously described [29].

### 2.3. Genotyping Cell Lines for p53 Status

Genotyping was executed using Sanger Sequencing at the VIB-Genetic Service Facility (Antwerp, Belgium) on PCR-amplified cDNA of the p53-coding region using primers: TTTCCACGACGGTGACACGCTTC and GGGAACAAGAAGTGGAGAATGTCAGTC as previously described [9].

### 2.4. In Silico Energy Calculations Using FoldX

Changes in thermodynamic stability due to a mutation were calculated as DDG (change in free energy, kcal/mol) using the molecular design toolkit FoldX (version 3b4 [30]). Side chains of the p53 structure were minimized (PDB code: 2AC0, Repair PDB command), and mutants were generated using the BuildModel command. The higher the value, the more destabilizing the mutation is.

### 2.5. SDS/Western Blot Analysis and Blue Native Page Analysis

Analysis of protein expression was carried out on different cancer cell lines plated at a density of 2.5 × 10^5^ cells/well in a 6-well plate at day 0. At day 1, cells were treated with control (DMSO), Foldlin or another indicated treatment as indicated at an active concentration for 16 h. At day 2, cells were washed in PBS and lysed in 200 μL NP40 lysis buffer (150 mM NaCl, 50 mM Tris-HCL pH 8, 1% IGEPAL (NP40)), containing a 1X complete protease inhibitor cocktail (Roche, Basel, Switzerland) and 1 U/μL Universal Nuclease (Pierce) for 30 min on ice. Lysates were subjected to Western blot analysis or blue native page analysis as previously described [9]. Antibodies for detection included anti-p53 DO1 (Santa Cruz Biotechnology, Dallas, TX, USA), anti-CHK1 (G-4, Santa Cruz Biotechnology) and anti-GAPDH (6C5; Santa Cruz Biotechnology). Quantification was performed via densitometry using the ImageJ software package. Normalization was performed compared to GAPDH levels of the input.

### 2.6. Meso Scale Discovery MULTI-ARRAY Microplate Assay (USA)

The MSD detection assay allows for the precise measurement of protein targets in small-volume samples via a sandwich immunoassay. The concentration of p53 was measured in NP40 lysates using the MSD platform carrying antibodies against total p53 (product #K15169D-1) or GAPDH (K151PWD-1). Detection was achieved based on electrochemiluminescence. The protocol was executed according to the manufacturer’s guidelines. Total p53 levels were normalized against the corresponding GAPDH signal.

### 2.7. SOD1 Transfection Experiments

HeLa cells were seeded at a density of 5 × 10^5^ cell per well in a 6-well plate. One day after seeding, cells were transfected with expression constructs for SOD1 WT, A4V or G93A, for the indicated times using the Lipofectamine 2000 transfection reagent (Thermo Fisher Scientific, Waltham, MA, USA) according to the manufacturer’s protocol. After transfection, cells were treated overnight (16 h) with 12.5 µM Foldlin. Cells were next lysed in 200 μL NP40 lysis buffer (150 mM NaCl, 50 mM Tris-HCL pH 8, 1% IGEPAL (NP40)), containing a 1X complete protease inhibitor cocktail (Roche) and 1 U/μL Universal Nuclease (Pierce) for 30 min on ice. Lysates were centrifuged for 15 min at 15,000 G, and resulting supernatants were collected as soluble fractions, while the insoluble fractions were dissolved in 8 M urea in PBS. Subsequent SDS/Western blotting was performed with the detection antibodies anti-myc tag and anti-GAPDH. Quantification was performed based on densitometry using the ImageJ software package. Normalization was corrected based on GAPDH levels of the input.

### 2.8. Immunofluorescent Staining and High Content Imaging Screening

Cells were plated in a 96-well plate (μells, Greiner) at a density of 8000 cells/well at day 0. At day 1, cells were treated at indicated doses for 16 h. Cells were fixed with 4% PFA (Thermo Scientific, Waltham, MA, USA, 16% PFA diluted in PBS) for 30 min and permeabilized and blocked in blocking buffer containing 1% Bovine Serum Albumine (BSA) and 0.2% Triton X100 in PBS for 1 h. Primary antibodies were added in blocking buffer at a dilution of 1:500 (DO1) and incubated overnight at 4 °C while shaking gently. Cells are washed 3 times for 5 min in PBS and subsequently incubated with DAPI (1PI ncu and/or secondary antibody (1:1000 goat-anti mouse alexa-594) in blocking buffer for 1 h. Imaging was performed on the InCell Analyzer 2000 (GE Healthcare, Chicago, IL, USA). The InCell Developer package (v1.9.2) allows the visualization, imaging and quantification of staining intensity and quantification of inclusions in cells following immunofluorescent staining. Statistical analyses were performed on a minimum of 1000 single cells/condition with R-studio (version 0.97.55) using R (3.0.1) software.

### 2.9. Gene Expression Analysis by Quantitative Real-Time PCR (qPCR)

RNA was extracted using the RNAeasy mini kit (Qiagen, Antwerp, Belgium) with on-column DNase treatment. cDNA was generated with the GoScript Reverse Transcription System (Promega, Leiden, The Netherlands) starting from 1 µg RNA. qPCR was run with the GoTaq Probe qPCR Master Mix (Promega Leiden, The Netherlands) on a CFX96 instrument (Bio-Rad, Temse, Belgium). Primer/probe sets were acquired from Integrated DNA Technologies (IDT, Leuven, Belgium), and sequences are highlighted in the table below. Samples were analyzed in triplicate, and fold-change analysis was performed using the Q-Base plus v3.3 analysis software (Biogazelle, Gent, Belgium) and R-Studio. 

### 2.10. Full Transcriptome RNA Sequencing

For each sample, the RNA concentration and purity were determined spectrophotometrically using the Nanodrop ND-1000 (Nanodrop Technologies, Wilmington, DE, USA), and RNA integrity was assessed using the Bioanalyzer 2100 (Agilent, Leuven, Belgium). Per sample, an amount of 1 µg of total RNA was used as input. Using the Illumina TruSeq^®^ Stranded mRNA Sample Prep Kit (protocol version: Part # 15031047 Rev. E—October 2013), poly-A containing mRNA molecules were purified from the total RNA input using poly-T oligo-attached magnetic beads. In a reverse transcription reaction using random primers, RNA was converted into first strand cDNA and subsequently converted into double-stranded cDNA in a second strand cDNA synthesis reaction using DNA PolymeraseI and RNAse H. The cDNA fragments were extended with a single ‘A’ base to the 3′ ends of the blunt-ended cDNA fragments, after which multiple indexing adapters were ligated, thereby introducing different barcodes for each sample. Finally, enrichment PCR was carried out to enrich those DNA fragments that had adapter molecules on both ends and to amplify the amount of DNA in the library. Sequence libraries of each sample were equimolarly pooled and sequenced on an Illumina NextSeq 500 instrument (High Output, 75 bp, Single Reads, v2) at the VIB Nucleomics core (www.nucleomics.be, accessed on 19 December 2022). RNA-seq reads were subsequently mapped to the human genome (Gencode v18) using STAR 2.5. Read counts per gene were obtained from the aligned reads using the htseq-count package (HTSeq 0.5.4p5). The Bioconductor/R package edgeR was used for normalization and differential gene expression analysis. Gene rankings based on the signed -log10 (FDR) values were used for Gene Ontology term enrichment analysis via Gorilla, following which, Revigo was used to analyze enriched GO terms. Up- and downregulated genes were identified using a cutoff of log2 fold change ≥ |1| and adjusted *p*-value ≤ 0.05. Upregulated genes were further analyzed using iRegulon v1.4 for the detection of upstream regulators. iRegulon analysis was performed with 19K motif collection and 1120 ChIP-seq track collection with putative regulatory regions defined as 20 kb centered around TSS.

### 2.11. Calculation of the Inhibitory Concentration of Foldlin

Cells were plated in a 96-well plate at 8000 cells/well at day 0. At day 1, cells were treated with Foldlin for 24 h at a concentration range between 0 and 50 μM (or as indicated in the figures/text). Cells were fixed with 4% PFA. Subsequent DAPI staining of nuclei and imaging via high content analysis allowed the counting of remaining nuclei after treatment at different concentrations. Statistical analysis to calculate IC_50_ values was performed using GraphPad Prism 6.0. Data were fitted to a non-linear regression three-parameter model.

### 2.12. Immunoprecipitation Experiments

NP40 lysates of control, Foldlin or MG-132 treated cells (M7449, Sigma Aldrich) were subjected to immunoprecipitation by adding the cell lysate to magnetic beads (Dynabead protein G, 1004D, Life technologies) coated with pAB240 (Abcam, 1 μg/IP reaction) or pAB1620 (Abcam, 1 µg/IP reaction) and incubated overnight at 4 °C. The beads were subsequently extensively washed in lysis buffer, and SDS/Western blot analysis was carried out, as previously described [9].

### 2.13. Proteasome Activity Test (Proteasome-Glo) and Caspase Glo Test

Cells were seeded in a white 96-well plate at an 8000 cells/well density and incubated overnight for attachment. Cells were treated with either DMSO control, MG-132 (1 µM), staurosporin (0.1 μM) or Foldlin (at IC_50_ concentration) for a total incubation time of 8 h (proteasome) or 16 h (caspase test). Proteasomal activity and caspase activity were monitored according to the manufacturer’s guidelines (ROS-Glo H_2_O_2_ assay, Promega, Leiden, The Netherlands; Proteasome-Glo, Promega, Leiden, The Netherlands, caspase-GLO 3/7 assay, Promega, Leiden, The Netherlands).

### 2.14. Screening of Enzo Library

HACAT cells were seeded at a density of 3000 cells/well in a volume of 20 μL/well and incubated for 24 h at 37 °C, with 5% CO_2_. After 24 h, compounds (or DMSO control) were added by an Echo Liquid Handler (Labcyte, San Jose, CA, USA) to a final concentration of 10 μM (0.5% DMSO), and cells were incubated for another 20 h. Afterwards, cells were fixed in 4% PFA for 15 min at room temperature and blocked and stained in PBS with 1% BSA and 0.2% TritonX-100 for 2 h at room temperature. Antibodies and dyes were as follows: anti-p53 (DO-1, Santa Cruz) 1:500; anti-HSP70 (4873S, NEB) 1:500; secondary antibodies Alexa Fluor 488 and DyLight 550, ThermoFisher, Antwerp, Belgium, 1:500; Hoechst 1:5000 and CellMask Deep Red 1 (ThermoFisher, Antwerp, Belgium), 1:2000. Cells were imaged at a magnification of 20× (water objective) on the Opera Phoenix system, and cell compartments (nucleus/cytoplasm/whole cell) were segmented by the Opera’s image analysis software using the DAPI and CellMask signal.

### 2.15. Statistical Analysis

All statistical analyses in this study were performed in R-Studio (version 0.99.878) using R (version 3.0.2).

## 3. Results

### 3.1. Pharmacological Induction of a Stress Response in Conventional and Primary Tumor Cell Lines

**Figure 1:**
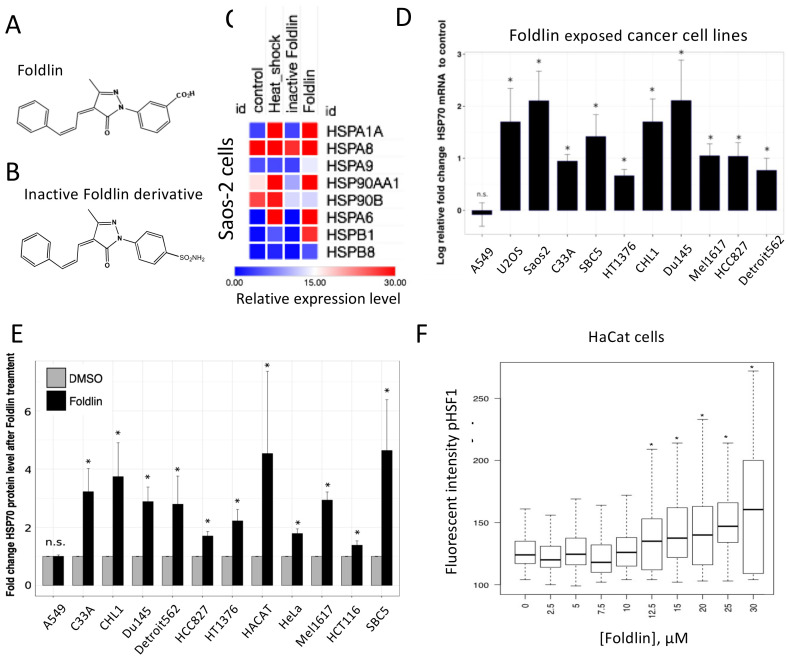
Foldlin and an inactive derivative induce a heat shock response in cancer cell lines. (**A**,**B**) Chemical structure of Foldlin: 3-(3-methyl-5oxo-4-((E)-3-phenylallylidene)-4,5-dihydro-1H-pyrazol-1yl) benzoic acid (B) or an inactive Foldlin derivative: 4-(3-methyl-5oxo-4-((E)-3-phenylallylidene)-4,5-dihydro-1H-pyrazol-1yl) benzenesulfonamide (C). (**C**) Heat map representation of a qPCR array to measure changes in mRNA expression levels of various heat shock proteins relative to GAPDH/β-Actin in DMSO control conditions or after exposure to heat shock (15 min at 45 °C), 10 µM Foldlin or its inactive derivative for 16 h. Fold-changes are indicated by the color code. (**D**) qPCR analysis of HSP70/HSPA1A mRNA levels of different cell lines after 16 h treatment with Foldlin (for each cell line at the IC_50_ concentration). Data are plotted as the log2 fold-change relative to the DMSO control ± SEM. Relative quantities normalized to the reference genes GAPDH and β-Actin, based on three independent repeats. (**E**) Meso Scale Discovery MULTI-ARRAY microplate assay for total HSP70 shows the fold-change in HSP70 protein levels following exposure to Foldlin (IC50 concentration for 16 h) relative to the DMSO control, normalized to GAPDH levels. The bar plot represents three independent repeats ± SEM). (**F**) High content analysis experiment showing boxplots of p-HSF-1 protein expression based on immunofluorescent intensity after exposure to Foldlin for 16 h in the HaCat cell line at the indicated concentrations. * *p* < 0.05, n.s. not significant.

As opposed to the well-described inhibition of various proteostatic components [13,31], in this study, we wanted to assess whether the activation of the cellular heat shock response would also lead to reduced levels of accumulated, mutant and/or aggregated p53 in tumor cells. To identify potential small molecule modulators of this system, we first tested 3-(3-methyl-5-oxo-4-((E)-3-phenylallylidene)-4,5-dihydro-1H-pyrazol-1yl) benzoic acid (further termed ‘Foldlin’), a previously identified small-molecule HSF-1 activator [32], and an inactive form (4-(3-methyl-5-oxo-4-((E)-3-phenylallylidene)-4,5-dihydro-1H-pyrazol-1yl) benzenesulfonamide) (Figure 1A,B and Supplementary Figure S1). Contrary to that with the inactive derivative, Foldlin was able to induce a broad-spectrum heat shock response in the Saos2 osteosarcoma cell line, which was comparable to an actual heat shock response at 45 °C (Figure 1C).

Subsequently, we selected a collection of established human cancer cell lines from various origins, of which, we previously characterized the p53 mutational, expression and aggregation status [9]. These included wild-type p53 (p53^WT^), natively folded but inactive contact mutant p53 (mutp53^CON^) and misfolded/aggregated structural p53 mutants (mutp53^STRU^), as well as a p53 null line (p53^NULL^) (Table 2, Supplementary Figure S2A) [7,9]. In these models, exposure to Foldlin increased the mRNA and protein expression of HSP70 (Figure 1D,E), with the exception of that in the multi-resistant A549 lung cancer cell line. Concurrent with this observation, the responsive HACAT cell line also exhibited a dose-dependent increase in HSF-1 activation, as depicted by increased levels of HSF-1 protein phosphorylation at serine residue 326 upon exposure to Foldlin (Figure 1F). Strikingly, the exposure of primary HUVEC cells to Foldlin (10 µM for 16 h) did not result in an upregulation of HSPA1A mRNA, suggesting that tumoral cells may be more sensitive to this compound than healthy primary cells (Supplementary Figure S2B).

Since conventional cell lines can sometimes divert from the original tumor from which they were derived, we also analyzed a panel of short-cultured, patient-derived primary glioblastoma (GBM) cell lines [33]. Moreover, here, we determined the p53 mutational, expression and aggregation status (Supplementary Figure S2C). Since these models are grown as non-adherent spheroid cultures, flow cytometry (FACS) was used to determine the ability of Foldlin to induce HSP70 protein expression—an observation that was made across multiple cell lines (Table 3, Supplementary Figure S2D). This shows that, despite their already enhanced HSF1 activation status, tumoral cells are still able to mount a classical heat shock response through HSF1 that results in the induction of HSP70 family members.

### 3.2. Pharmacological Activation of HSF1 Results in Reduced Levels of Misfolded and Aggregated mutp53

**Figure 2:**
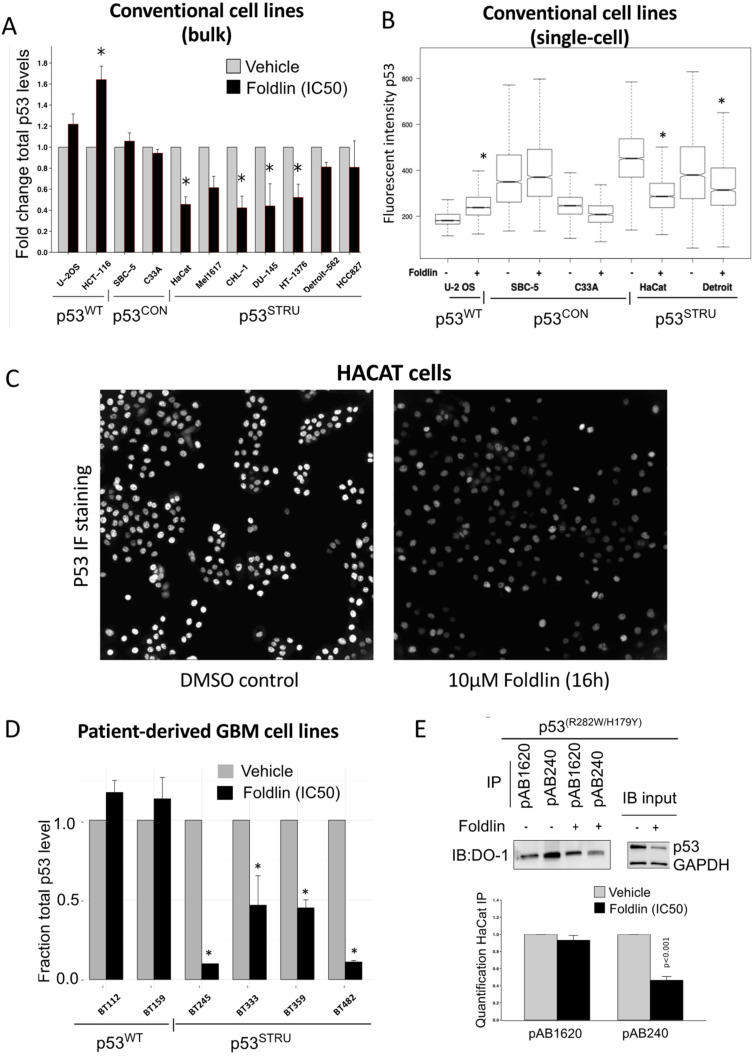
Reduced protein levels of structurally destabilized mutant p53 in cancer cell lines upon exposure to Foldlin. (**A**) Total p53 protein level quantification after a 16 h exposure to Foldlin at the IC50 concentration using an MSD/ELISA assay with an array of established tumor cell lines. Total cellular p53 levels are normalized to cellular GAPDH levels, and the fraction of the total p53 protein level after treatment is represented in comparison to a DMSO vehicle control. The experiment was executed based on *n* ≥ 2 independent repeats. Data are represented as means ± SEM). Statistical analysis performed via one-way ANOVA/TUKEY (HSD). * *p* < 0.05. (**B**) Boxplot representation of single-cell p53 fluorescent intensity measurements via high-content image analysis in DMSO control and Foldlin-treated conditions at the IC_50_ concentration for 16 h based on a subset of cell lines. *n* > 1000 cells/condition. (**C**) Immunofluorescent images of HaCat cells treated overnight with DMSO or Foldlin (10 μM). P53 was detected via DO-1 staining (white) and imaged via a high-content imaging set-up at 10× magnification. (**D**) MSD/ELISA analysis for the total p53 protein level based on different glioblastoma cell lines after overnight DMSO vehicle or Foldlin treatment at the IC5O concentration. Total p53 levels are normalized to total GAPDH levels. *n* ≥ 2 independent repeats. (**E**) Conformational immunoprecipitation experiments (IP) with p53 conformational-specific antibodies (pAB1620 recognizing well-folded p53 DNA-binding domain, pAB240 recognizing unfolded/mutant p53 DNA-binding domain) after Foldlin treatment (IC50 concentration, 16 h treatment), and the input levels (immunoblot (IB)) of both p53 and GAPDH via SDS/Western blot. Upper panel: representative SDS/Western blot of the HaCat cell line, with below quantification of p53 protein levels. Plot shows the mean of three independent repeats +SEM; statistical analysis was performed via Student-*t* testing.

Next, we assessed the p53 levels and their aggregation status following exposure to Foldlin (10 µM for 16 h). Using ELISA and co-immunoprecipitation based assays, we found that Foldlin treatment resulted in reduced levels of misfolded mutp53^STRUC^ protein, while leaving natively folded mutp53^CON^ levels largely unaffected. Exposure to Foldlin also resulted in a mild increase in p53^WT^ levels, which can in part be explained by the previously reported ability of Foldlin to function as a weak MDM2 inhibitor [34] (Figure 2A). These “bulk” findings were also confirmed at the single-cell level using high-content measurements of p53 levels (Figure 2B,C) which also showed the time-dependent activity in HACAT cells (Supplementary Figure S2E). Importantly, these observations were similar in both conventional and patient-derived cell lines (Figure 2D).

**Figure 3:**
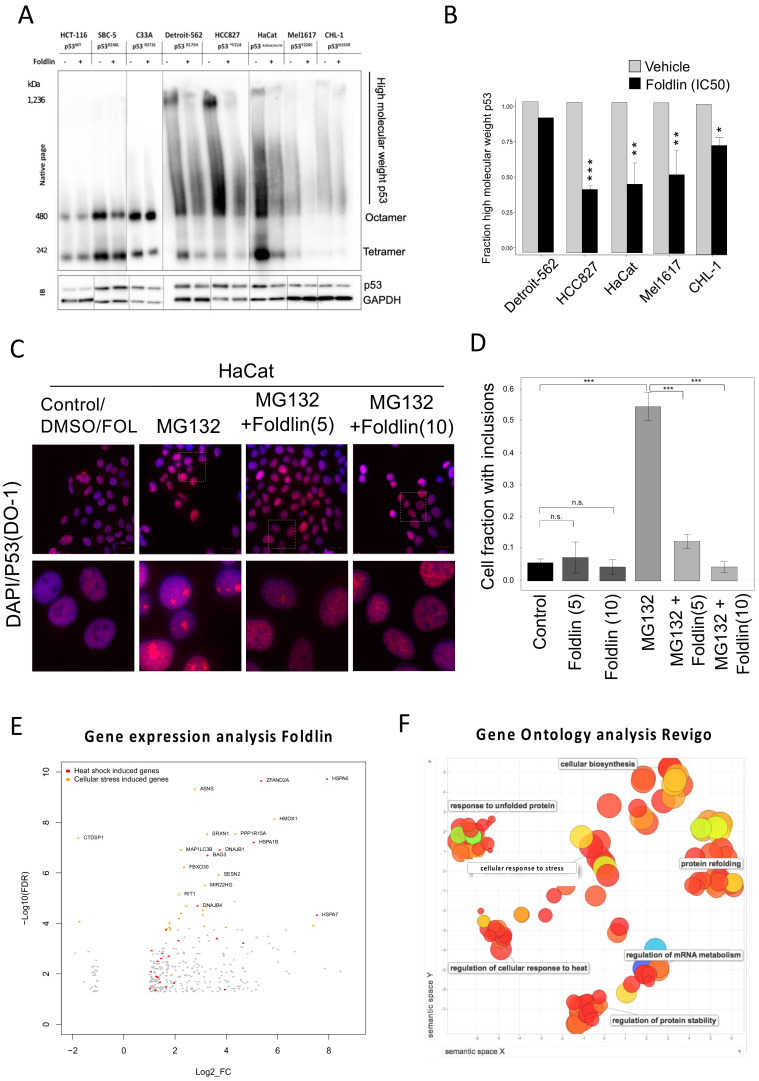
Exposure to Foldlin reduces p53 protein aggregation and nuclear inclusion body formation by inducing a cellular heat shock response. (**A**,**B**) Blue native page analysis (**A**) of p53 of cell lysate vehicle (DMSO) or Foldlin treatment (IC50 concentration, 16 h) based on different cell lines. One representative blot out of two or more repeats. (**B**) Quantification of high molecular weight p53 in the native page experiment (>425 kDa) normalized to GAPDH input levels, *n* ≥ 2. The plot indicates the mean ± SEM. Statistical analysis was performed via a Student’s t test, * *p* < 0.05, ** *p* < 0.01, *** *p* < 0.001., n.s. not significant. (**C**,**D**) Immunofluorescent images (**C**) of HaCat cells treated overnight with DMSO vehicle, MG132 (1 μM) or Foldlin (5 or 10 μM) in combination with MG132 (1 μM) treatment. P53 was detected using DO-1 staining (red) and imaged by a high content imaging set-up at 60× magnification. (**D**) Quantification of the number of cells showing inclusions caused by overnight Foldlin treatment alone (5 or 10 μM), MG132 treatment (1 μM) alone or that in combination with Foldlin (5 μM or 10 μM). Bar plot represents the mean ± SEM) of three independent repeats. Statistical analysis was performed via an ANOVA-Tukey(HSD); *p*-values are shown on the bar chart. (**E**,**F**) Transcriptome profiling (**E**) across eight cancer cell lines following which differential gene expression analysis was performed (represented by the log2 fold change (Log2_FC) and a false discovery rate (FDR) < 0.05). (**F**) Gene Ontology analysis of the genes significantly altered by Foldlin across eight cancer cell lines.

The striking specificity of Foldlin to reduce mutp53^STRUC^ was further highlighted in the HACAT cell line, which harbors both a mutp53^STRUC^ and a mutp53^CON^ alleles (p53^R282W^ and p53^H179Y^, respectively) [9]. In this cell line, we performed immunoprecipitation experiments using conformational p53 antibodies recognizing either unfolded/misfolded (pAB240 antibody) or natively folded (pAB1620 antibody) p53 DNA-binding domains. Upon Foldlin treatment, the p53–pAB240 fraction was decreased, while the p53–pab1620 fraction remained largely unaffected (Figure 2E), an observation that was also seen in DU145 cells, confirming Foldlin’s ability to reduce structurally affected mutant p53 in diverse cellular backgrounds (Supplementary Figure S2F). In addition, native PAGE (NPAGE) analysis also indicated a significant decrease in high molecular weight fractions of p53, suggesting that Foldlin also affected the stability of aggregated p53 in the exposed cancer cell lines (Figure 3A,B).

Finally, we determined the cytotoxic profile of Foldlin in each of the used models. This showed that Foldlin is cytotoxic when given for >48 h at IC_50_ values between 1 and 15 µM across the various models (Table 1 and Table 2). Strikingly, even though the IC_50_ values were largely consistent across the various models, we found that Foldlin induces caspase 3/7-dependent apoptosis in cell lines expressing mutp53^STRUC^ or functional p53^WT^ more strongly than that in mutp53^CON^ or p53^NULL^ lines (Supplementary Figure S2G).

### 3.3. Pharmacological Activation of a Heat-Shock Response Mediated by Foldlin Prevents the Formation of Nuclear Inclusion Bodies of p53 upon Proteostatic Stress

As previously described in HACAT cells, the inhibition of proteasomal activity by MG132 results in the formation of nuclear inclusion bodies [9]. The fluorescent immunocytochemistry staining of untreated or DMSO-treated HACAT cells (p53^R282W/H197Y^) using the DO-1 monoclonal antibody showed diffuse nuclear p53 staining in the absence of visible inclusion body formation, indicative of soluble, oligomeric p53, as described before [9]. In contrast, upon proteasomal inhibition mediated by MG132 in HaCaT cells, mutp53 formed visible nuclear inclusion bodies resembling the phenotype we previously observed in patient samples carrying aggregated mutp53 [9]. The combined exposure of tumor cells to Foldlin and MG132 resulted in the absence of nuclear inclusions (Figure 3C,D), suggesting that the induction of a cellular heat shock response was sufficient to enhance protein homeostasis and prevent nuclear inclusion body formation. Importantly, Foldlin did not function as a proteasomal inhibitor (Supplementary Figure S2H)

### 3.4. Mapping the Proteostatic Network Induced by Foldlin

**Figure 4:**
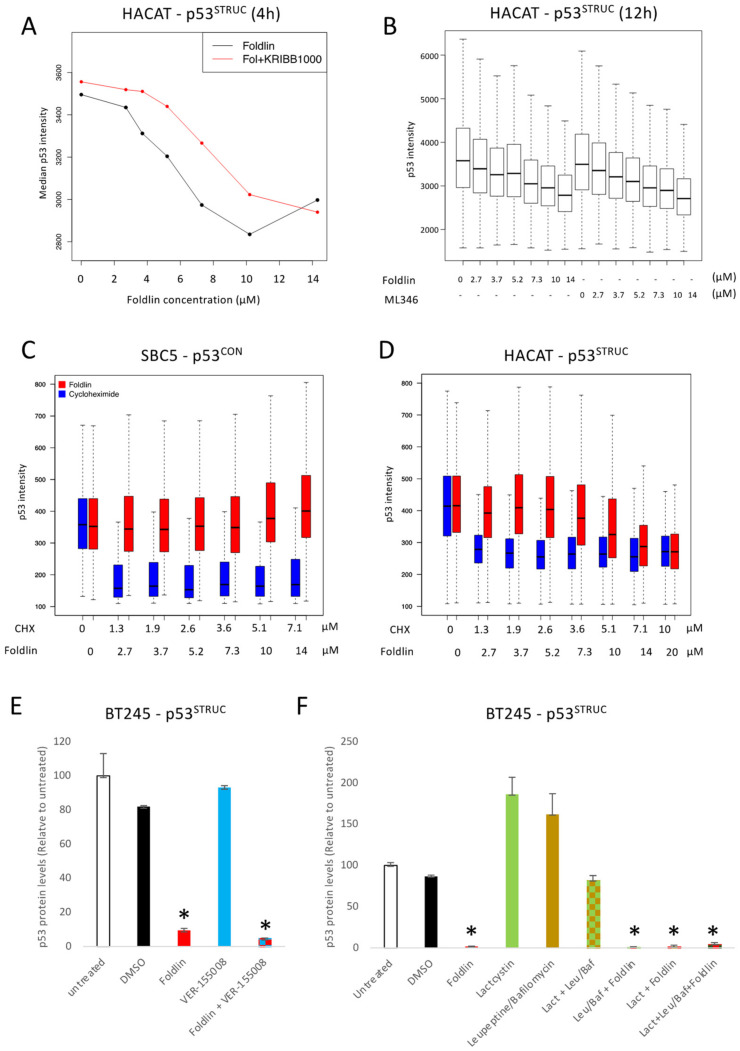
Mechanistic analysis of Foldlin in cell lines containing contact or structurally destabilized mutant p53. (**A**,**B**) High-content analysis quantification of HACAT cells being exposed to Foldlin or Foldlin and KRIBB11, an HSF1 inhibitor (**A**), or ML346, an HSF1/HSP70 inducer (**B**). (**C**,**D**) Boxplot representation of p53 intensity levels following high-content imaging at the single-cell level (>1000 cells/condition) in SBC5 (**A**) or HaCaT (**B**) cells. Exposure to cycloheximide (blue) or Foldlin (red) is compared. (**E**,**F**) p53 protein levels measured using MSD ELISA following exposure to the indicated compounds and treatments as indicated in the manuscript. For the combined treatments with Foldlin, only small numbers of cells remained for which the p53 levels were measured. Results are shown for three independent repeats ± SEM. * *p* < 0.05, n.s. not significant.

To further map the molecular pathways activated by Foldlin, we performed bulk transcriptome analysis using RNA sequencing on eight tumor cell lines (A549, C33A, CHL1, DU145, HaCaT, HCC827, HT1376 and MEL1617; Table 1) in control and Foldlin-exposed conditions. This led to the identification of 310 common genes that were significantly upregulated and 27 genes that were downregulated upon exposure to Foldlin, with a log2-fold-change larger than 1 and false discovery rate (FDR) ≤ 0.05 (Figure 3E, Supplementary Table S1). Functional analysis revealed that Foldlin induces a broad cellular stress response, as defined by the upregulation of genes involved in the heat shock, oxidative stress, genotoxic stress and ER stress response. Related to the heat shock response, this set included nine chaperones and 13 co-chaperones with mainly HSP70 family members and their co-chaperones being strongly induced (highlighted in Figure 3E), as expected from the original screen that identified Foldlin [32]. In addition, Gene Ontology (GO) term enrichment analysis (using Gorilla) revealed the ‘unfolded protein response’, ‘protein folding’ and ‘response to heat’ as significantly enriched terms. Clustering of the GO term enrichment using REVIGO and Gene Set Enrichment Analysis (GSEA) confirmed these findings (Figure 3F). We then analyzed the gene set using iRegulon, a tool that predicts the transcription factors for which activity can explain the majority of the differentially expressed genes based on known or calculated transcription factor-binding sites. This identified HSF-1, 2 and 4, as well as ATF-3 as primary drivers of the observed changes in expression profiles, confirming major proteostatic remodeling mediated by the compound, but also found the proto oncogene FOS and the p53-homolog p73, highlighting the potentially more pleiotropic nature of Foldlin. Subsequent profiling of responsive genes via Ingenuity Pathway Analysis (IPA) confirmed the proteostatic modulating activity of Foldlin as it upregulates an HSF-1-dependent proteostatic network, including HSP70 and HSP90 family members and their cochaperones (HSP40, BAG3). Independent proteomics analysis of a p53^null^ cell line (SAOS2), as well the mutp53 lines (DU145 (p53^P223L/V274F^) and HACAT (p53^R282W/H179Y^)), confirmed the strong induction of HSP70 (HSPA1A) and its closely related HSPA6 family member (not shown). In addition, we also observed the accumulation of HSP110 [35] and DNAJB1 [36], both of which have been shown to mediate protein disaggregation in conjunction with HSP70 family members. The role of HSF1 activation was further strengthened by the use of KRIBB11 (a small molecule HSF1 inhibitor [37]) and ML360 (another HSF1/HSP70 inducer [38]). First, the combined treatment of HACAT cells with KRIBB11 (1000 nM) and Foldlin led to a delayed reduction in p53 expression levels at 6 h (Figure 4A). This was primarily observed at the lower concentrations of Foldlin (between 4–6 µM), where the applied levels of KRIBB11 were not too toxic yet, but still sufficient to block the induced activity of HSF1. In addition, the applicability of HSF1 inducers to remove excessive p53 was further confirmed by applying ML360 to HACAT cells, where it also led, similar to Foldlin, to a reduction in p53 protein levels (Figure 4B).

In addition, the accumulation of SQSTM1/p62 in response to Foldlin treatment, both at the RNA and protein level (Supplementary Figure S3A), also suggests the possibility that autophagy might be enhanced in response to Foldlin treatment [39]. Finally, various genes involved in the oxidative stress response, such as HMOX1 [40], were also significantly upregulated.

### 3.5. Mechanistic Analysis of Foldlin-Mediated Changes in p53 Protein Levels

The observed reduction in p53 levels upon the induction of a heat shock and more general stress response by Foldlin urged us to further investigate the underlying mechanisms that caused this change in protein levels. This was performed by using various additional tool compounds.

#### 3.5.1. Foldlin Does Not Interfere with RNA Transcription or Protein Translation

Here, we assessed whether Foldlin affects the overall mRNA levels of p53, in addition to its subsequent translation to p53 protein. First, we investigated p53 mRNA levels following exposure to Foldlin, where we did not observe a significant change in p53 mRNA levels (Supplementary Figure S3B for HACAT cells). Second, to investigate potential interference with p53 translation, we compared the changes in p53 protein levels in cells treated with the translation inhibitor cycloheximide (CHX; dose range applied for 16 h). There, we observed that a large fraction of p53 protein levels was reduced by blocking translation. However, when comparing p53^CON^ with p53^STRUC^ mutations, we observed that p53^CON^ mutations were affected by CHX but not Foldlin, whereas p53^STRUC^ could be reduced to the same extent by either CHX or Foldlin (Figure 4C,D). Moreover, the clear induction of the de novo translation of HSP70 proteins (and many other chaperones) shows that Foldlin does not act as a general translation inhibitor.

#### 3.5.2. Foldlin Does Not Depend on HSP70 Effector Functions

We investigated whether the direct upregulation and activation of HSP70 family members was linked to the altered mutp53 protein levels. BT245 GBM cells were pre-treated for 4 h with VER-155008 (1 mM), a pan-HSP70 family inhibitor [41], following which Foldlin was added for another 16 h. This showed that the inhibition of HSP70 was insufficient to block the reduction in the observed p53 levels (Figure 4E). In addition, the overexpression of HSPA6, one of the primary induced HSP70 family members upon exposure to Foldlin, was unable to alter the p53 protein levels within 72 h (not shown).

#### 3.5.3. Foldlin Does Not Depend on HSP90 Effector Functions

From the original HSF-1 activator screen in which Foldlin was identified [32], potential leads were counter-screened for HSP90 and proteasomal inhibition. p53 mutant stabilization in tumor cells can be due to its interaction with HSP90, safeguarding it from MDM2 and CHIP E3 ligase activity and subsequent proteasomal degradation [15]. Consequently, mutp53 degradation can be induced by HSP90 inhibition [13].

To exclude HSP90 inhibition mediagted by Foldlin, we assessed checkpoint kinase 1 (CHK1) protein levels upon exposure to Foldlin or Ganetespib (a well-described HSP90 inhibitor [13]), via SDS/Western blotting (Supplementary Figure S3C). CHK1 is an obligatory HSP90 client protein of which abundance is strictly dependent on HSP90 activity [42]. We observed that the CHK1 protein levels were comparable between the control and Foldlin-exposed conditions, while Ganetespib reduced CHK1 levels. This confirms that Foldlin does not inhibit HSP90 activity.

#### 3.5.4. Foldlin Activity Does Not Depend on Proteostatic Degradation, Autophagy or Shedding

We confirmed that Foldlin does not directly inhibit proteasomal activity (Supplementary Figure S2G), similar to that in previous reports [43,44]. However, the reduced p53 levels could still result from altered proteasomal activity upon exposure to Foldlin. To assess this hypothesis, tumor cells were treated with a proteasomal inhibitor, lactacystin [45], either alone or in combination with Foldlin. While the blocking of proteasomal activity increased p53 protein levels, the combination of Foldlin with lactacystin did not prevent reduced p53 protein levels (Figure 4F). Alternatively, autophagy and/or chaperone-mediated autophagy (CMA) have been identified as other mechanisms of aberrant p53 removal from cells [46]. As described above, we observed an accumulation of the autophagic marker SQSTM/p62 upon exposure to Foldlin. This suggests that autophagy might be involved in the reduction of mutp53 levels. Similar to blocking the proteasome, blocking autophagy and/or CMA using a mixture of Leupeptine/Bafilomycine also increased levels of p53 protein, suggesting that also this mechanism is involved in p53 protein homeostasis. However, even though we observed increased protein levels of p62 (Supplementary Figure S3A) upon exposure to Foldlin, the combined exposure to autophagy inhibitors and Foldlin did not prevent Foldlin from reducing detectable p53 protein levels (Figure 4F). This strongly suggests that both autophagy and proteasome-mediated protein degradation are required but are largely redundant. Unfortunately, the combined exposure to both proteasomal and autophagic inhibitors was extremely toxic to the cells, precluding us from drawing meaningful conclusions from those experiments. Finally, exosomal shedding was recently highlighted as another mechanism for a cell to remove unwanted proteins from its cell body. When measuring the p53 levels in the supernatant from control or Foldlin-treated cells, we did not observe an increase in p53 protein levels (not shown).

### 3.6. Reduced Levels of Misfolded and Aggregated SOD1 Variants upon the Pharmacological Activation of HSF1

**Figure 5:**
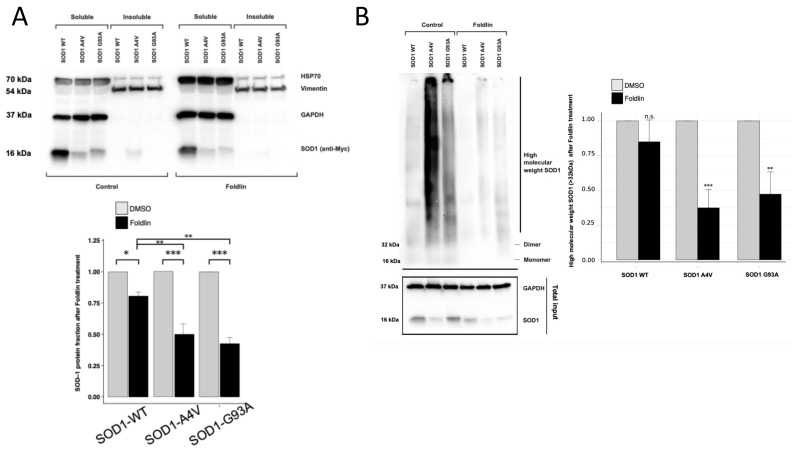
Biological activity of Foldlin in cell lines containing SOD1 mutant proteins. (**A**) (Upper figure) Representative SDS/Western blot of SOD-1 (wild-type, A4V or G93A)-transfected HeLa cells. Shown are soluble and insoluble fractionation of HSP70 and SOD-1 after DMSO control or Foldlin treatment (12.5 µM, 16 h after initial 6 h construct transfection). Vimentin and GAPDH were used as a loading control for the insoluble or soluble fraction, respectively. One representative blot out of three independent repeats. (Below) SDS/Western blot quantification of the total SOD1 protein level in transfected HeLa cells for WT SOD1 and SOD1 mutants A4V and G33A after Foldlin treatment (12.5 µM, 16 h after initial 6 h construct transfection). The total SOD1 levels were calculated as the sum of the soluble and insoluble fractions, as shown based on the SDS/Western blot above. All results show five independent repeats ± SEM. (**B**) Representative blue native page analysis blot of SOD1 wild-type, A4V and G93A mutant constructs transfected in HeLa cells shows treatment with Foldlin (12.5 μM, 16 h) and its effect on the high-molecular smear of SOD1 (>32 kDa). Total input levels of the blot are shown below (GAPDH and SOD1). One representative blot out of three repeats. (Right) Quantification of the intensities of the SOD1 high-molecular smear (>32 kDa) of the blue native page blots. Results are shown for three independent repeats ± SEM. * *p* < 0.05, ** *p* < 0.01, *** *p* < 0.001, n.s. not significant.

Next, to assess the ability of Foldlin to affect the levels of other disease-associated aggregated proteins, we also transfected HeLa cells with wild-type superoxide dismutase 1 (SOD1), as well as the G93A and A4V SOD1 mutants, the aggregation of which occurs in a subset of familial cases of amyotrophic lateral sclerosis (ALS), where the mutant proteins form cytotoxic inclusions in the cytoplasm of motor neurons [47]. We confirmed that Foldlin exposure induces HSP70 in the transfected cells and impacts HSF1 activation based on the degradation of the various SOD1 species. Similar to our observation for p53, aggregated SOD1, but not wild type SOD1, levels were reduced upon exposure to Foldlin (Figure 5A,B). This demonstrates that a stress response induced by Foldlin is capable of reducing the levels of misfolded and aggregated proteins in a more general way.

The combined data highlight that the molecular mechanism of Foldlin cannot be pinpointed to one single mechanism, but that redundant pathways are at play to reduce p53 protein levels upon the induction of a cellular heat-shock-like stress response, including the concerted activation of both proteasome and autophagy to remove p53 from the cells.

### 3.7. Phenotypic Screen for p53 Level Modulators

**Figure 6:**
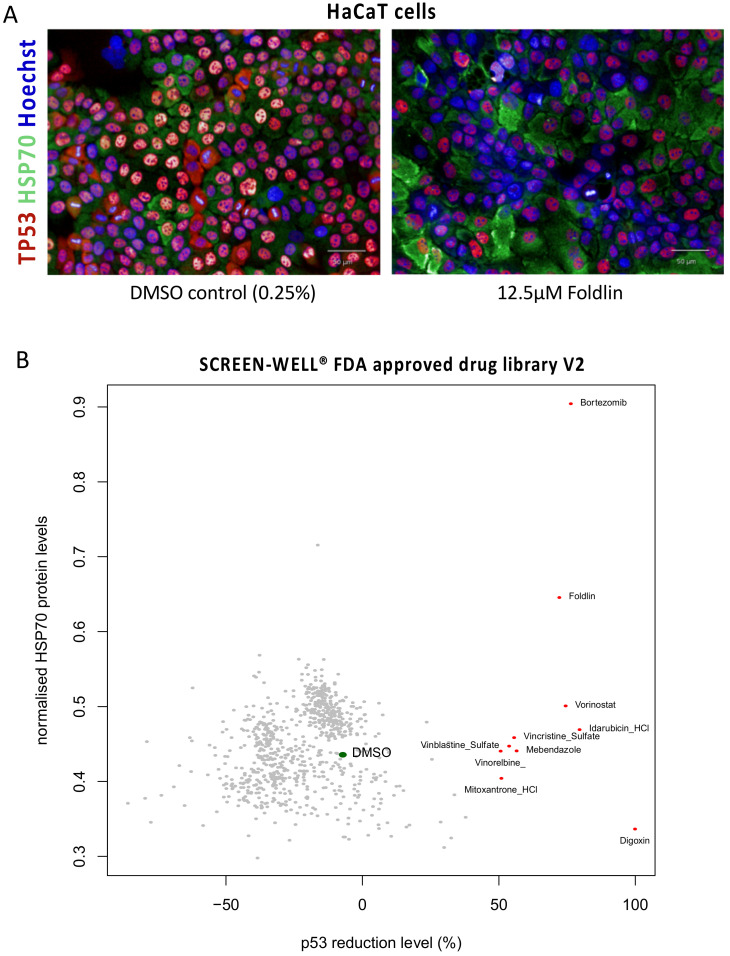
High-content screening for molecules reducing misfolded p53 levels while measuring HSP70 levels in HaCaT cells. (**A**) Immunofluorescent images of HaCat cells treated overnight with DMSO or Foldlin (12.5 μM). P53 and HSP70 levels were detected using immunofluorescent staining (blue = Hoechst nuclear dye; red = TP53 protein; green = HSP70 protein. (**B**) Dotplot representation indicating the level of the p53 protein level reduction (x-axis) vs. the induction of HSP70 protein levels (y-axis). Molecules with >50% reduction in p53 levels are indicated with a red dot. The average effect of exposure to the DMSO control condition is shown by the green dot.

Given the difficulty in mapping the mode-of-action of Foldlin and considering the strong effects it exerts on misfolded/aggregated p53, we performed a phenotypic screen to search for molecules with similar effects. Therefore, a phenotypic, high-content-based screenable assay using the HACAT cell line was developed. Here, the p53 and HSP70 protein levels were measured using immunofluorescent staining following compound exposure with Foldlin exposure/treatment as a positive control (Figure 6A). Screening was performed using a repurposing compound library (SCREEN-WELL^®^ FDA approved drug library V2, Enzo life sciences), which consists of 778 FDA-approved compounds. These were selected to maximize chemical and pharmacological diversity and screened for their potential activity to reduce p53 levels and their effect on HSP70. Apart from Foldlin, this assay identified only nine additional molecules that significantly reduced p53 levels (>50% reduction compared to the control treated condition; Supplementary Table S2). The most effective molecule was the previously identified Digoxin [48], which nearly depleted p53 protein levels completely (>99%), compared to the 72% reduction induced by Foldlin. However, it is highly toxic and blocks the translation of multiple proteins, including p53. Regardless, this molecule did not affect HSP70 levels, in contrast to Bortezomib, a known proteasomal inhibitor currently used for the treatment of lymphoma/myeloma in the clinic [49], which shows a similar pattern to Foldlin, combining both p53 reduction and HSP70 induction (Figure 6B). Other molecules that achieved significant reductions in p53 protein levels included the HDAC inhibitor Vorinostat, the DNA topoisomerase inhibitors Idarubicin and Mitoxantrone, and several microtubule-destabilizing agents (Mebendazole and the vinca alkaloids Vinblastine, Vincristine and Vinorelbine), albeit with a mild induction of HSP70 levels (Figure 6B, Supplementary Table S2). It remains to be seen whether these molecules have similar effects on p53 homeostasis in patients upon their administration.

## 4. Discussion

Mutp53 aberrantly accumulates in a large fraction of tumors [50]. While well-folded p53 acts a tumor suppressor, mutp53 acquires oncogenic gain-of-function properties, promoting tumor growth, metastasis and drug resistance [51]. The dependence of tumors on mutp53 is often such that tumor cells appear addicted to the presence of elevated levels of mutant p53 protein and that the genetic knock-out of mutp53 results in cell death, improving the response to other treatments [18]. Despite decades of research, the mechanisms leading to oncogenic gain-of-function activity of mutp53 still remains only superficially understood and is probably diverse [51,52].

The mechanisms behind mutp53 accumulation are being unraveled and consist of several layers of complexity. p53 loss-of-function results in the impairment of its self-regulating degradation by the MDM2 E3-ligase leading to its accumulation in the cell. While in cells containing a transcriptionally active wild-type p53 gene such accumulation of p53 leads to the activation of senescence and apoptosis pathways, cells containing mutant p53 accumulate p53 protein without the induction of these pathways, allowing this protein, present at a supranatural concentration, to acquire altered and oncogenic activities. One such altered activity is linked to the conformational properties of mutp53, which can contribute to the exacerbation of its accumulation in tumor cells, effects that are further amplified by affecting the conformational properties of wild-type p53 (which may still be present as well), as wild-type p53 is a thermodynamically unstable protein requiring HSP90 interaction for its efficient folding. Indeed, while nascent wild-type p53 only transiently interacts with HSP90, misfolded structural p53 mutants establish more stable interactions thereby hijacking HSP90 from its normal client proteins (such as wild-type p53), but also preventing the actual proteasomal degradation of the aberrant protein. As HSP90 binds to and stabilizes near-native hydrophobic pockets of nascent client proteins, this probably explains why structural missense mutations are so common in p53. [53]. Third, we and others have shown that the sustained accumulation of misfolded mutp53 results in p53 aggregation in tumors and that aggregation itself contributes to the gain-of-function activity of mutp53 [7,54]. However, aggregation is an important modulator of the biological stability of mutp53 as these proteolytic-resistant entities affect the mechanisms of degradation available to mutant p53. While monomeric misfolded mutp53 is degraded by Hsp70-assisted CHIP-mediated proteasomal degradation (which is facilitated by Hsp90 inhibition [55]), aggregates cannot easily enter the proteasomal channel. In addition, aggregated p53 further contributes to the impairment of proteasomal degradation by blocking the 20S channel. This limits the clearance of misfolded mutant p53 and excludes the proteasomal degradation of aggregated p53. This observation has been confirmed in neurodegenerative diseases where Hsp90 inhibition precludes aggregate formation. In the same disease models, preformed aggregates can be partially degraded by Hsp70-dependent autophagy, while large and mature aggregates are often too stable to be degraded even by macro-autophagy [25].

The activation of the heat shock response in tumors goes, at first glance, against previous studies showing that chaperone upregulation is beneficial for tumor malignance and that many tumors display chaperone addiction. This is also the reason why chaperone inhibitors have been explored as a valuable therapeutic option in cancer [56]. Indeed, studies using HSF1-knockout mice showed a strong reduction in cancer incidence, and it is now widely accepted that the heat shock response is, to some extent, elevated in cancer cells. Modulating the proteostasis network in cancer cells by inhibiting actors of the HSF-1 pathway, such as HSP90 or the HSF-1 protein itself, is an intensely studied approach in cancer treatment [57]. However, as cancer cells reside in a state of intermediate expression of HSPs, protecting them from, for instance, environmental stress, the strategy of overexpressing chaperones in cancer cells can provide a way to alleviate the inhibition of important tumor suppressor proteins. Moreover, it was shown that the transcriptional program of HSF1 in malignancy fundamentally differs from the HSF1 program induced by thermal stress [28] and is more akin to the HSF1 profile observed during development [58]. It has become clear that HSF-1 exhibits distinct transcriptional programs upon different types of activation of the protein (e.g., phosphorylation, acetylation, sumoylation, etc.) during the stress response, resulting in a distinct transcriptional program during cancer development in comparison to that with physical heat stress [28]. For example, the HSPA6 protein is upregulated during thermal heat stress, but not during tumor progression.

Overall, as highlighted in the introduction, several strategies have already been designed to deal with aggregated p53 species. In this work, we observed that the induction of a heat shock-like stress response in cancer cells resulted in the efficient removal of pathologically aggregated mutant p53 and as such represents yet another way to deal with pathological protein accumulation through aggregation. Interestingly, the activation of the chaperone machinery prevented the formation of p53 nuclear inclusion bodies, for which we have previously shown that they are linked to a worse clinical outcome in colon cancer and glioblastoma [9]. Strikingly, this approach seemed to also be effective at reducing protein levels of the aggregation-prone mutant SOD1, a protein that aggregates in motoneurons in the context of ALS.

The mode of action of how Foldlin achieves this effect in cells still remains elusive, however. While transcriptional profiling identified the induction of a heat shock-like response, in addition to a more general stress response, we were unable to pinpoint Foldlin’s activity to a specific cellular activity. Rather, our data suggest that this molecule induces a combination of stress responses, which act in a highly redundant way. In non-pathological conditions, misfolded proteins are generally degraded by either the proteasomal degradation machinery or through autophagy [59]. As described above, the activity of these pathways is challenged in pathological conditions, including in cancer cells. Blocking either of these two major protein degradation pathways, however, seemed insufficient to undo the effects of Foldlin, further highlighting the redundant activity of both pathways. In addition, Foldlin did not seem to affect protein translation or to be fully dependent on HSP70 or HSP90 activity. Strikingly, cancer cells seemed to be more susceptible to this induction than HUVEC cells [60]. Whether this relates to the ability of HUVEC cells to remove this compound via enhanced pump activity, similar to our observation of Foldlin resistance in A549 lung tumor cells, remains to be investigated.

From a chemical point of view, both Foldlin and ML346 contain reactive groups (pyrazole, benzoic acid) that may act as Michael acceptors and as such interfere with multiple biological processes, such as disulfide bond generation [61]. It has been suggested that compounds with such reactive groups may not have a suitable therapeutic window in clinical applications. Considering the striking biological activity of Foldlin in combination with the difficulty to map Foldlin’s activity based on (a) specific pathway(s), we also sought to determine whether other molecules could be identified with similar activities. By screening a repurposing library of 778 FDA-approved compounds in a phenotypic assay, we found that the proteasomal inhibitor Bortezomib also led to significant reductions in misfolded p53, in combination with the induction of a heat shock/HSP70 response.

Bortezomib (Velcade^®^) is a dipeptide drug that blocks the 26S proteasomal unit and is currently approved for the treatment of multiple myeloma and mantle cell lymphoma [62]. In either disease, the tumor cells seem to be highly dependent on proteasomal activity, but generally still require an additional insult to induce apoptosis, explaining why Bortezomib is always used in combination with other cytotoxic drugs. Part of the activity of Bortezomib has also been attributed to the activation of wild-type p53 [63] or its homologue p73 [64] to induce cell death. Strikingly, p73 is known to become inactivated by aggregated p53 [7], and its reactivation seems to be in line with the ability of bortezomib to reduce misfolded/aggregated p53. Finally, the combination of Bortezomib with HDAC inhibitors also seemed to be an effective strategy to target tumor cells containing GOF mutant p53 [65]. In the same line, the effect of Bortezomib on cancer cells containing mutant p53 required an additional insult to effectively induce apoptosis [66]. It remains to be seen whether the application of bortezomib on top of other cytotoxic drugs in the context of a tumor containing an aggregated p53 mutant will lead to a higher efficacy to target tumor cells. As such, even though Foldlin may not have a sufficiently wide therapeutic window, the concept of inducing a cellular heat-shock-like stress response to deal with misfolded proteins, being it in a cancerous or neurodegenerative context, may still be worth pursuing.

## Figures and Tables

**Table 1:** qPCR primer and probe sequences.

Name	Forward Primer	Reversed Primer	Probe
HSPB8	AAAGATGGATACGTGGAGGTG	GGGAAAGTGAGGCAAATACTG	/56-FAM/CTGGCAAAC/ZEN/ATGAAGAGAAACAGCAAGA/3IABkFQ/
HSP90AA1	GTCTGTGAAGGATCTGGTCATC	CAGCAGTAGGGTCATCTTCATC	/56-FAM/AGACAGGAG/ZEN/CGCAGTTTCATAAAGCA/3IABkFQ/
HSP90B1	AAACGGGCAAGGACATCTC	AAACCACAGCAAGATCCAAAAC	/56-FAM/TCAGCGGGT/ZEN/GTCTGGGATTAATTTCAA/3IABkFQ/
HSPB1	ATGTCAACCACTTCGCCC	GTGAAGCACCGGGAGATG	/56-FAM/AGATCACCG/ZEN/GCAAGCACGAGG/3IABkFQ/
HSPA1A	AGGACATCAGCCAGAACAAG	CTGGTGATGGACGTGTAGAAG	/56-FAM/CTGCGAGAG/ZEN/GGCCAAGAGGAC/3IABkFQ/
HSPA6	AAGCAGACCCAGACTTTCAC	TCTCACCCTCATACACCTGG	/56-FAM/CACCTACTC/ZEN/GGACAACCAGCCT/3IABkFQ/
HSPA8	GGACAAGAGTACGGGAAAAGAG	GTCCCTCTGCTTCTCATCTTC	/56-FAM/TTCAATGTC/ZEN/TTCCTTGCTCAAACGGC/3IABkFQ/
HSPA9	GTATTCTCTACTGCCGCTGATG	TTCAATCTGAGGAACTCCACG	/56-FAM/TCTCCAGCC/ZEN/ATCTCTCTTTCACCCT/3IABkFQ/
TP53	AATACTCCACACGCAAATTTCC	CAAGCAGTCACAGCACATGA	/56-FAM/CCTCCTCAG/ZEN/CATCTTATCCGAGTGGA/3IABkFQ/
ACTB	CCTTGCACATGCCGGAG	ACAGAGCCTCGCCTTTG	/56-FAM/TCATCCATG/ZEN/GTGAGCTGGCGG/3IABkFQ/
GAPDH:	TGTAGTTGAGGTCAATGAAGGG	ACATCGCTCAGACACCATG	/5HEX/AAGGTCGGA/ZEN/GTCAACGGATTTGGTC/3IABkFQ/

**Table 2:** Panel of cell lines with endogenous p53 status and Foldlin IC_50_ values.

Cell Line	Tumor Type	P53 Genotype	Protein Classification	Aggregational Status(bNativePAGE)	Mean IC50 (±SEM)(μM)
U-2 OS	Osteosarcoma	WT	Wild type	No	12.31 ± 1.8
A549 *	Lung carcinoma	WT	Wild type	No	>50
HCT-116	Colorectal carcinoma	WT	Wild type	No	10.87 ± 1.6
SAOS-2	Osteosarcoma	Null	Null	/	13.07 ± 2.6
HeLa	Epithelial cervix adenocarcinoma	null	Null	No	11.73 ± 1.5
SBC-5	Lung carcinoma	R248L	Contact mutant	No	15.88 ± 1.3
C-33A *	Retinoblastoma	R273C	Mixed conformation	Yes	13.94 ± 1.4
HT-1376 *	Bladder carcinoma	P250L	Structural mutant	Yes	>50
CHL-1 *	Melanoma	H193R	Structural mutant	Yes	6.67 ± 1.4
DU 145 *	Prostate carcinoma	P223L/V274F	Structural mutant	Yes	17.24 ± 1.4
MEL1617 *	Melanoma	Y220C	Structural mutant	Yes	8.28 ± 1.7
HaCat *	Immortalized keratinocyte	R282W/H197Y	Structural mutant	Yes	11.15 ± 1.4
HCC827 *	Lung carcinoma	^V218	Structural mutant	Yes	8.17 ± 1.5
Detroit-562	Pharyngeal carcinoma	R175H	Structural mutant	Yes	23.5 ± 1.9

Table adapted from De Smet et al.; * RNASEQ-analyzed cell lines.

**Table 3:** p53 mutation status of 11 short-cultured patient-derived glioblastoma cell lines.

GBM Cell Line	p53 Mutation Status	IC_50_ Foldlin (μM)
BT112	WT	5.02
BT569	WT	/
BT164	Q164 *	10.84
BT271	WT	/
BT239	WT	5.71
BT333	V173M	6.68
BT359	C275Y	14.09
BT482	Fs152	8.93
BT245	R249S	/
BT607	WT	7.22
BT159	WT	1.97

* Stop codon, Fs: frameshift; /: not determined.

## Data Availability

Data and cell lines can be obtained upon request.

## Supplementary Materials

The following are available online at https://www.mdpi.com/article/10.3390/cells12060960/s1; Table S1: Differential gene expression analysis using bulk RNA sequencing of eight cells lines in control and Foldlin-treated cell lines (10 µM); Table S2: High content screening of 778 FDA-approved compounds with the maximal effect on p53 and HSP70 levels; Figure S1: Synthesis and QC of Foldlin; Figure S2: Aggregational status of cell lines and Foldin-induced heat-shock analysis; Figure S3: Molecular analysis of the mode of action of Foldlin.
