# Supplementary material for: Reduced Levels of Misfolded and Aggregated Mutant p53 by Proteostatic Activation
Source: Cells. 2023 Mar 21;12(6):960. doi: 10.3390/cells12060960 (PMC10047295; doi:10.3390/cells12060960)
Supplement: Supplementary file 1 [file cells-12-00960-s001.zip › cells-1558792-supplementary/supplementary files/Figures.pdf]

Supplementary Figure 1

A

Synthesis

3-(3-methyl-5-oxo-4-((*E*)-3-phenylallylidene)-4,5-dihydro-1H-pyrazol-1-yl)benzoic acid:

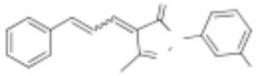

The second double bound might still be a mixture of isomers.

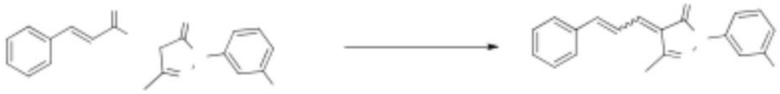

Molecular Formula: $C_{20}H_{16}N_2O_3$

Molecular Weight: 332,353 g/mol

Quantity: 51 x 100 mg

Appearance: red solid

Structural Analysis Data:

Apparatus	Name Spectrum	Purity (%)
LC-MS	VIB007-001-aa-11	97 % (UV, 254 nm)
NMR	VIB007-001-aa-11	OK

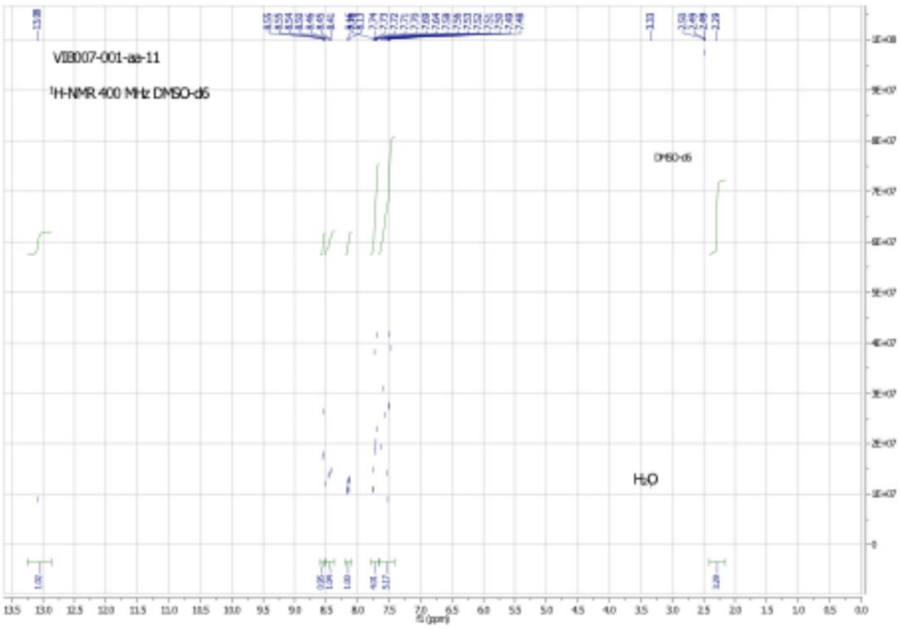

Figure S1. Details on the synthesis of Foldlin by Ecosynth. Given is the structure, NMR and MS data showing purity and quality.

Supplementary Figure 2

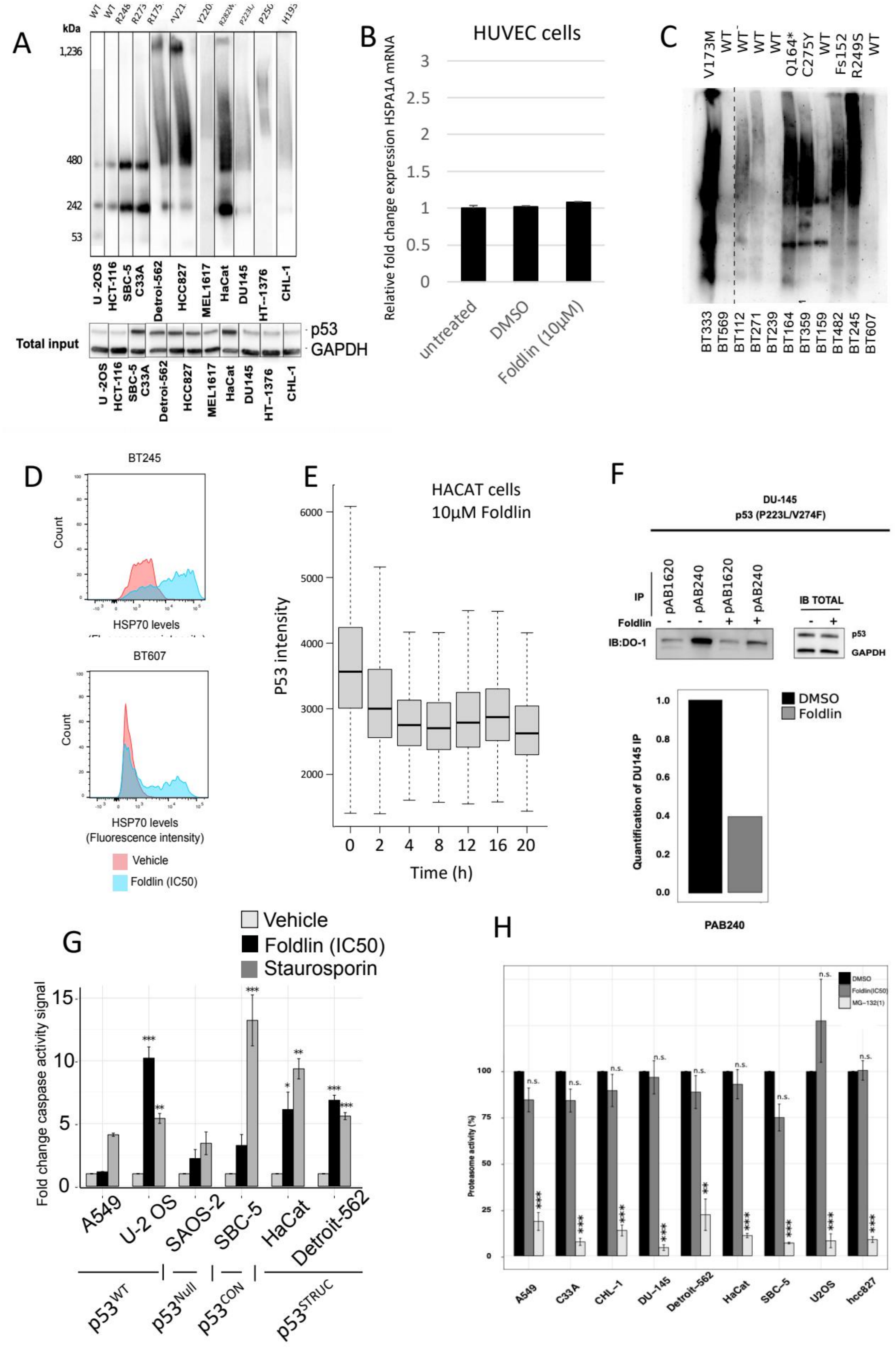

Figure S2. A,C, Blue Native page characterization of different cancer cell lines
(conventional (A) or patient derived GBM cell lines (B)) shows

discrimination between natively folded, tetrameric p53 and aggregated species of p53 by the formation of a high molecular weight smear on the PAGE gel. Under the native blot are the input levels shown by SDS/Western blot for (total) p53 (DO1) and GAPDH [9]. One representative blot for each cell line out of >3 repeats. **B,** Quantitative realtime qPCR experiment to measure HSP70 mRNA levels in HUVEC cells exposed to DMSO or Foldlin (10μM). **D,** Flow cytometry analysis on GBM cell lines in control and Foldlin treatment conditions at IC_{50} concentration for 16 h. **E,** Quantification of the high content screening analysis to determine the time-course effect of Foldlin on the p53 protein levels in HACAT cells. **F,** Conformational immunoprecipitation experiments with p53 conformational specific antibodies (pAB1620 recognizing WT, pAB240 recognizing unfolded/mutant) after Foldlin treatment (IC50 concentration, 16 h treatment) and the input levels of both p53 and GAPDH by SDS/Western blot. Upper panels show SDS/Western blot, with below quantification of protein levels of the DU145 cell line. **G,** Caspase 3/7-GLO apoptosis assay on different cell lines after overnight DMSO vehicle, Staurosporin (0.1 μM concentration) or Foldlin treatment at IC_{50} concentrations. Represented is the fold change of Relative Luminescent Units (RLU) normalized to vehicle DMSO control. Staurosporin was used as positive control for this assay, as staurosporin is a very potent inhibitor of protein kinases and often used as a general control for apoptosis induction. **H**, Cell-based proteasome activity assay (Proteasome-GLO, Promega), executed on different cell lines, with overnight exposure of cells to proteasomal inhibitor MG-132 (1 μM, overnight) or Foldlin alone (IC_{50} concentration). Three independent repeats. Statistical analysis by student's t test to DMSO control. ** $p<0.01$, *** $p<0.001$, n.s. : not significant.

Supplementary Figure 3

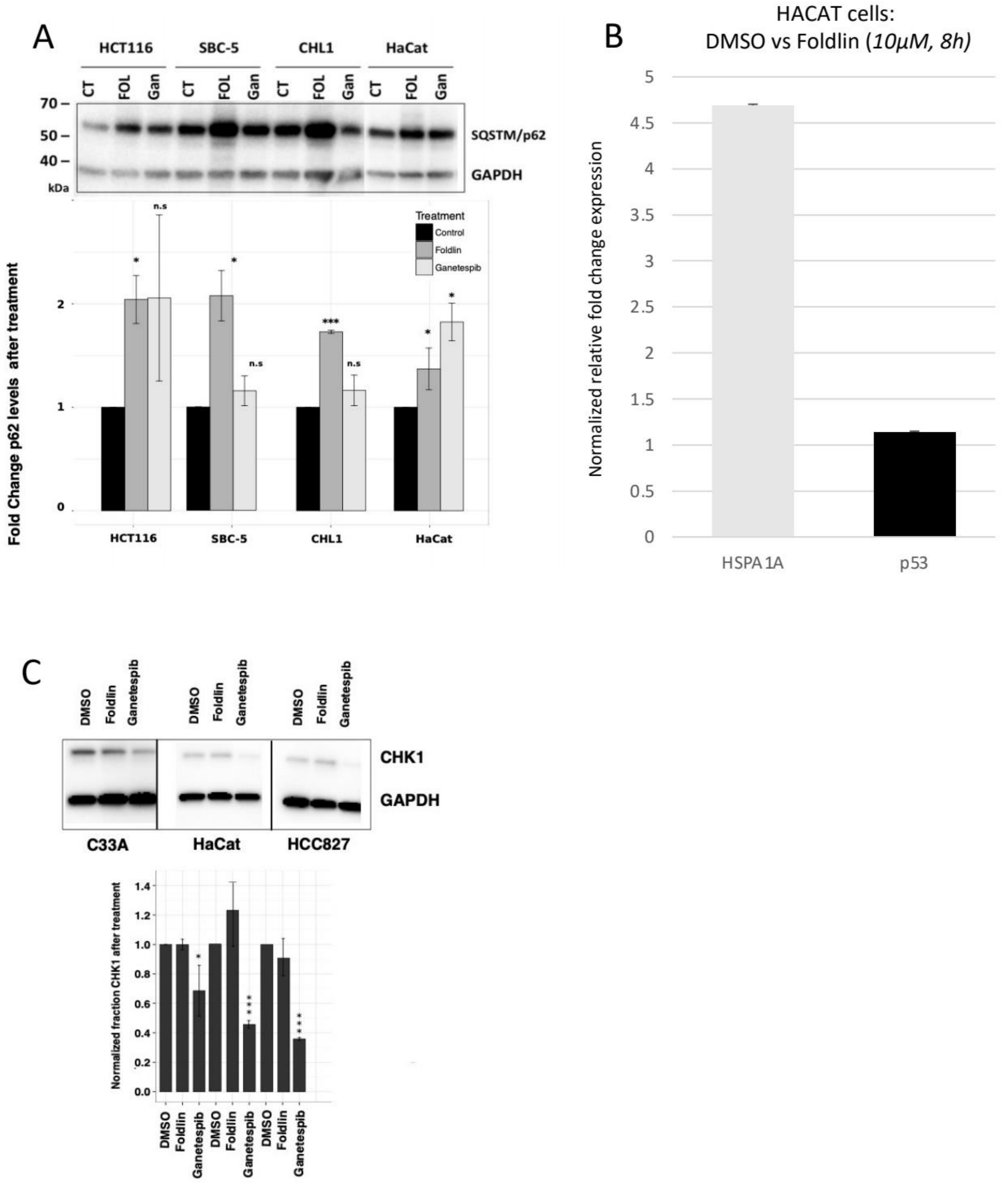

Figure S3. A, Representative SDS/Western blot of SQSTM/p62 levels shows p62 expression levels after overnight Foldlin treatment (IC_{50} concentrations) or Ganetespib (500 nM) in different cell lines. Below is

the quantification of p62 levels in comparison to DMSO control. Three independent repeats. *$p<0.05$, **$p<0.01$, ***$p<0.001$, n.s.: not significant. statistical analysis done by Student's t test. **B**, qPCR experiment to estimate the relative changes in mRNA expression of HSPA1A and TP53 in HACAT cells following exposure to Foldlin. **C,** Representative SDS/Western blot of HSP90 client protein CHK1 levels in different cell lines before and after treatment. Degradation of CHK1 is indicative of HSP90 inhibition. Treatment of cell lines is done overnight, Ganetespib at 500 nm and Foldlin at IC_{50} concentration. Below the blot is the quantification of the repeats. Barplot represents means of three independent repeats +-SEM. Statistical analysis by student's t test to DMSO control. **$p<0.01$, ***$p<0.001$, n.s. : not significant.
